# Effect of Air-Particle-Abrasion Protocols on Surface Roughness and Early Biofilm Formation of Zirconia

**DOI:** 10.3290/j.ohpd.a44321

**Published:** 2020-02-12

**Authors:** Vanessa Cruz Macedo, Priscilla Cristoforides Pereira, José Renato Cavalvanti de Queiroz, Amanda Maria de Oliveira Dal Piva, Dayanne Monielle Duarte Moura, Rubens Nisie Tango, Marco Antonio Bottino, Rodrigo Othávio de Assunção e Souza

**Affiliations:** a Professor, Department of Dentistry, Universidade de Braz Cubas, Mogi das Cruzes, SP, Brazil. Experimental design, performed the experiments, wrote the manuscript.; b Dentist, Department of Dental Materials and Prosthodontics, São Paulo State University (Unesp), Institute of Science and Technology, São José dos Campos/SP, Brazil. Experimental design, performed the experiments, wrote the manuscript.; c Professor, Department of Biotecnology, UnP – Laureate Universities, Natal, Brazil. Experimental design, performed the experiments, wrote the manuscript.; d PhD Student, Department of Dental Materials and Prosthodontics, São Paulo State University (Unesp), Institute of Science and Technology, São José dos Campos/SP, Brazil. Wrote the manuscript, contributed substantially to discussion.; e PhD Student, Department of Dentistry, Federal University of Rio Grande do Norte (UFRN), Brazil. Wrote the manuscript, contributed substantially to discussion.; f Adjunct Professor, Department Dental Materials and Prosthodontics, São Paulo State University (Unesp), Institute of Science and Technology, São José dos Campos/SP, Brazil. Idea, hypothesis, proofread the manuscript.; g Professor, Department Dental Materials and Prosthodontics, São Paulo State University (Unesp), Institute of Science and Technology, São José dos Campos/SP, Brazil. Idea, hypothesis, contributed substantially to discussion.; h Adjunct Professor, Department of Dentistry, Prosthodontic Division, Federal University of Rio Grande do Norte (UFRN), Natal/RN, Brazil. Idea, experimental design, proofread the manuscript.

**Keywords:** blasting, scanning electron microscopy, surface roughness, zirconia

## Abstract

**Purpose:**

The air-particle-abrasion on zirconia in the gingival area of connectors and pontics in fixed partial dentures appears to increase fracture resistance. This study evaluated ‘in situ’ biofilm formation on the zirconia surface after different air-particle-abrasion protocols.

**Materials and Methods:**

Ninety sintered blocks (5 × 5 × 2 mm) of yttrium partially stabilised zirconia (Y-TZP) were obtained and randomised among nine groups according to the factors ‘type of particle’ (Alumina 50 and 110 µm; Cojet and Rocatec) and ‘pressure’ (2.5 and 3.5 bar) used for sandblasting for 10 s. The surface roughness (Ra/Rz) was measured before and after sandblasting. For the in-situ analyses, custom-made removable intraoral devices n = 10 with one sample of each group attached to the buccal area were used by volunteers for 8 h at night. The specimens were analysed under confocal microscopy to quantify both biovolume and thickness of the initial biofilm formed. One-way analysis of variance (ANOVA) and Dunnett’s tests were performed (5%).

**Results:**

The roughness values ranged from 0.05 to 0.39 µm for Ra and from 0.35 to 2.11 µm for Rz, p = 0.00. Mean biofilm thickness ranged from 0.06 and 0.54 µm (p = 0.005), while the biovolume values were between 0.02 and 0.61 µm^3^/µm^2^ (p = 0.002). Values statistically significant for biofilm thickness and biovolume were found in groups sandblasted with Rocatec using 3.5 bar.

**Conclusion:**

In order to increase the fracture resistance of zirconia fixed partial dentures (FPDs), the air particle abrasion of zirconia with SiO_2_ (110 μm/3.5 bar), in the gingival area of connectors and pontics, should be avoided.

The use of ceramics in dentistry has enabled the fabrication of aesthetic restorations with satisfactory clinical performance, as established in the literature.^7,26^ Among the ceramics, zirconia partially stabilised by yttria (Y-TZP) has been highlighted due to its toughness, particularly appropriate for making infrastructure crowns and fixed partial dentures (FPDs).^12,31^

When FPDs are subjected to occlusal loads, tensile^32^ stress occurs, mainly in the gingival area of the connectors, which can promote cracking and subsequent fracture of the substructure.^24^ Due to biocompatibility of the material, it is possible to expose the zirconia substructure in the lower region of the connectors. This procedure does not affect aesthetics and saves space (approximately 0.7–1.0 mm), since it is not necessary to apply the ceramic covering in this region.^12^

It has been reported that the blasting of zirconia in the gingival area of FPD connectors and pontics creates a layer of compressive stress due to phase transformation at room temperature, and consequently increases the fracture resistance of infrastructure prostheses with a zirconia ceramic base.^13^ This layer of compressive stresses must be overcome by a crack in order to propagate, explaining the greater fracture toughness of zirconia.^13^ Sandblasting with particles of alumina (45 μm) was recommended to be avoided because it decreased the resistance of the FPD zirconia.^5^ While the alumina particles coated with silica (30 μm), strength was maintained. However, this procedure promotes an increase in the surface roughness at this region, which favours oral biofilm formation and thus the presence of secondary caries and periodontal problems. This initial adhesion of oral bacteria to tooth structure or restorative material is considered a critical step in the biofilm formation that can cause tissue or mineral damage in tooth-supporting structures. Also, mature biofilm formed in larger quantities appears to occur more rapidly on surface-roughened compared with polished surfaces,^2,8,21^ and its mechanical removal is hindered. However, this scenario is unknown with basted zirconia ceramics. Air-particle-abrasion is also applied to improve bond strength between zirconia and resin cements.^28^ However, inappropriate air-abrasion can generate microcracks that could decrease fracture resistance.^5,24^ Alumina (Al_2_O_3_) promotes more retentive surfaces^9^ and Al_2_O_3_/SiO_2_ modifies the surface, improving adhesion to the silane.^3,19^ Alumina and alumina coated with silica (Al_2_O_3_/SiO_2_) are especially used to air blast the zirconia surface.^2,33^ Several studies evaluated different sandblasting protocols involving the size and type of particles, pressure, distance and time of blasting on mechanical properties as bond, fatigue and fracture strengths.^17,23,28,33^ Nowadays, there is still no universal protocol to improve the success of zirconia restorations.^5,17,28^ It is well known that airborne particles can increase zirconia surface roughness; also, that high roughness values are related to oral biofilm formation^2,8^ and the type of material also plays a role, notably zirconia, that has a less homogeneous surface compared with other materials resulting from the sintering process,^8^ and can compromise the restorations success with the possibility of caries and periodontal diseases.^15^

Therefore, the present study evaluated the effects of different sandblasting protocols on surface roughness and initial ‘in-situ’ biofilm formation. The hypotheses were that the sandblasting favour early biofilm formation and that larger particles at higher pressure increase the biofilm biovolume and thickness.

## Material and Methods

### Sample Preparation

Ninety ceramic blocks (Zirconia Cercon, Dentsply Ceramco, Burlington, NJ, USA) were cut and sintered according to the manufacturer’s recommendations, to obtain the final dimensions of 5 mm × 5 mm × 2 mm. The ceramic surfaces were polished with 400-, 600-, and 1200-grit sandpaper (3M Brazil, Campinas, Brazil) under water cooling and randomly distributed among nine groups (n = 10) in accordance with the ‘sandblasting protocol’ shown in Table 1.

**Table 1:** Groups distribution according to ‘sandblasting protocol’. Brand names, manufacturers and materials used in this study

Group	Sandblasting protocol	Brand name	Manufacturer
Control	–	–	–
Al_2_O_3_50/2.5	Aluminium oxide (50 µm) Pressure: 2.5 Bar	Aluminium Oxide (# 320)	Polidental Ind. e Com Ltda
Al_2_O_3_50/3.5	Aluminium oxide (50 µm) with Pressure: 3.5 Bar		
Al_2_O_3_110/2.5	Aluminium oxide (110 µm) Pressure: 2.5 Bar	Aluminium Oxide (# 100)	
Al_2_O_3_110/3.5	Aluminium oxide (110 µm) Pressure: 3.5 Bar		
SiO_2_30/2.5	Aluminium oxide (30 µm) coated silica Pressure: 2.5 Bar	Cojet System	3M ESPE/ Irvine, CA, USA
SiO_2_30/3.5	Aluminium oxide (30 µm) coated silica Pressure: 3.5 Bar		
SiO_2_110/2.5	Aluminium oxide (110 µm) coated silica Pressure: 2.5 Bar	Rocatec System	
SiO_2_110/3.5	Aluminium oxide (110 µm) coated silica Pressure: 3.5 Bar		

The ceramic blocks were embedded in 10% isopropyl alcohol and subjected to ultrasonic cleaning (Vitasonic, VITA Zahnfabrik, Bad Säckingen, Germany) for 10 min. The samples were sandblasted with a microetch (Microjato Standard, Bio-Art, San Carlos, Brazil) for 10 s. For standardisation of the distance between the device tip and the ceramic (10 mm), a metallic device was used to position and fix the samples during sandblasting.

### Surface Roughness Analysis

The surface roughness was analysed before (i = initial) and after the sandblasting. A single precalibrated examiner performed quantitative analysis of surface roughness using profilometry (Mitutoyo SJ 400, Tokyo, Japan), with a cut-off of 3 mm. The roughness parameters analysed were Ra and Rz, with Ra corresponding to the arithmetic average of the absolute values of the ordinates of removal (peaks and valleys) in the midline within the measurement path and Rz is the arithmetic average of the five highest peaks and the five deepest valleys. Three measurements were made on each sample surface, with a distance of 3 mm between samples. An average value was obtained for each sample (three readings in different directions), after which the arithmetic mean for each group was calculated.

### Early Biofilm Formation Analysis

#### Intraoral device

For the in-situ study, 10 volunteer graduate students from São Paulo State University, Institute of Science and Technology (ICT Unesp) of both genders with an adequate standard of oral hygiene (no signs of caries or periodontal disease) and no change in salivary flow were selected. The clinical examinations involved assessment of the VPI (visible plaque index) and of the GBI (gum bleeding index). Those who had habits related to smoking and alcohol, who used drugs that interfere with salivary secretion, and who had used antibiotics in the 3 months prior to baseline were excluded.^14^

Volunteers were informed of the survey and agreed to participate by signing an informed consent document. The research project was submitted to the Ethics Committee (CEP) of the ICT Unesp, in full compliance with the provisions of Resolution No. 196/96 of the National Health Council.

**Fig 1:**
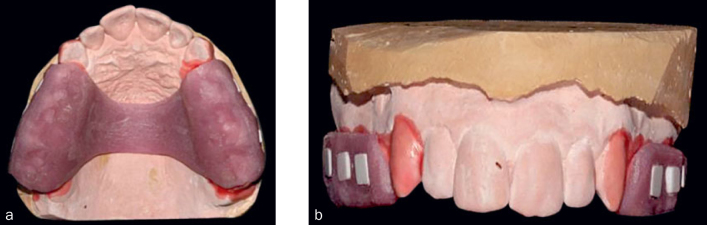
Intraoral resin photoactivated device with ceramic samples fixed on the buccal area: (a) occlusal view, (b) front view.

Moulds were made of the volunteers’ mouths (Jeltrate, Dentsply Ind. e Com., Petrópolis, RJ, Brazil), and dental plaster models (maxillary and mandibular) were obtained (Dentsply Ind. e Com.). Individual devices were fabricated from photoactivated resin (Elite LC Tray, Zhermack, Rovigo, Italy), covering the crowns of the molars and premolars, regions that irrespective of arch, demonstrated higher accumulations of dental plaque than anterior regions.^29^ After resin adaptation, ceramic samples were placed in the buccal region on the device (six on the right side and five on the left) to form niches, which were subsequently fixed. The oral device, which covered the crowns of the molars and premolars, was photoactivated in unity EDG-Lux (400–500 mW/cm^2^ for 7 min). Occlusal adjustment was performed with the teeth in habitual occlusion, with a ribbon used to establish the occlusal contact points and the positions of centric relation and eccentric movements (Fig 1a, b).

The samples were then fixed to the device with cyanoacrylate (Superbond, Loctite, São Paulo, Brazil). The device was disinfected with 1% sodium hypochlorite for 10 min. Before intraoral use, participants performed their usual oral hygiene, but without the use of toothpaste, to avoid interference of antimicrobial substances in initial biofilm formation.^4^ To evaluate the biofilm formed on the samples, participants used the oral device for 8 h during sleep, which corresponds to the time for initial formation of plaque and its proliferation.^6^ No food or drink was consumed during use of the device.

A piece of double-sided adhesive tape was bonded to a sterile, disposable petri dish (90 mm x 15 mm) (Prolab, Curitiba, PR, Brazil). The samples were removed from the device carefully, so that the surfaces to be analysed were facing up, and were transported stably.

#### Confocal laser scanning microscopy (CLSM) for biofilm analysis

Specimens removed from the oral device were stained with a commercial Live/Dead Bacterial Viability and Counting kit (Invitrogen Molecular Probes, Eugene, OR, USA) according to the manufacturer’s recommendations. This kit consists of two dyes, SYTO 9 (green), which identifies living cells, and propidium iodide (red), which stains dead cells.

Dyes were provided by means of a single-channel automatic pipette (volume from 0.5 to 10 μl) (HTL Labmate, Warsaw, Poland) at a proportion of 4 µl of dye to 1 µl of sterile saline, and each was dispensed into a sterile Eppendorf microtubule. One drop of each solution was dispensed onto the samples. The action time of the dye was 15 min in the dark, according to the manufacturer’s recommendations.

Blocks were placed on glass coverslips and analysed by confocal laser scanning (LSM 510 META, Zeiss, Oberkochen, Germany). The samples were then placed on a glass coverslip, with the surface to be analysed left in contact with it, to facilitate biofilm analysis. The wavelength of light used for excitation of the dye was 488 nm, and all light emitted from 500 to 550 nm and below 560 nm was collected by different filters. Optical lenses were used with increasing 10/0.3× to preview the entire sample, and with increasing 63/0.3× biovolume to analyse the average thickness of the biofilm for quantification via COMSTAT software (The MathWorks, Natick, MA, USA). For this analysis, there were ‘stacks’^20^ from each interface area for each analysed specimens for forming 3D images, and the number of optical sections varied depending on the thickness of the biofilm accumulated on the different groups of samples (average of 0.8 µm).

### Scanning Electron Microscope (SEM) Analysis of Surface Roughness

Surface roughness was analysed qualitatively after sandblasting in SEM to characterise each tested group (Model Inspect-S50, FEI Company, Hillsboro, OR, USA), with acceleration speed of 20 kV up to approximately 1000×.

### Statistical Analysis

For values of initial and final roughness (Ra/Rz µm), biovolume (μm^3^/μm^2^), and average thickness (µm), one-way analysis of variance (ANOVA) and Dunnett’s tests were performed, with a confidence interval of 5%.

## Results

Statistical assumptions were evaluated before statistical analysis. The results indicated that the results were normally distributed, and, plotted against predicted values, the uniformity was checked; therefore, no ANOVA assumptions were violated.

### Surface Roughness

The mean surface roughness values after surface treatments ranged from 0.05 to 0.34 µm for Ra and from 0.35 to 2.11 µm for Rz parameters. One-way ANOVA showed that initially Ra-i (p = 0.391) and Rz-i (p = 0.862) were not statistically significant different among groups. However, the evaluated sandblasting protocols significantly influenced the average depth roughness of Rz (p = 0.001) and Ra (p = 0.001). Moreover, Dunnett’s test revealed that all groups treated presented similar roughness (Ra and Rz) means that were higher compared with those of the control group (Table 2).

**Table 2:** Means and standard deviations of initial and final surface roughness values, biovolume, and average biofilm thickness accumulated, and homogeneous groups from the Dunnett analysis

Groups	Ra initial (µm)	Ra final (µm)	Rz initial (µm)	Rz final (µm)	Biovolume (µm^3^/µm^2^)	Average thickness (µm)
Control	0.05 ± 0.02	0.05 ± 0.02^a^	0.34 ± 0.15	0.34 ± 0.15^A^	0.02 ± 0.01^1^	0.15± 0.05^‡^
Al2O350/2.5	0.05 ± 0.02	0.14 ± 0.02^a^	0.34 ± 0.15	0.97 ± 0.11^B^	0.26 ± 0.31^1^	0.23 ± 0.30^‡^
Al2O350/3.5	0.05 ± 0.01	0.24 ± 0.09^b^	0.38 ± 0.11	1.55 ± 0.69^B^	0.16 ± 0.05^1^	0.18 ± 0.11^‡^
Al2O3110/2.5	0.05 ± 0.02	0.21 ± 0.10^b^	0.42 ± 0.19	1.43 ± 0.63^B^	0.26 ± 0.26^1^	0.22 ± 0.23^‡^
Al2O3110/3.5	0.11 ± 0.17	0.33 ± 0.10^b^	0.42 ± 0.20	2.11 ± 0.56^B^	0.04 ± 0.04^1^	0.06 ± 0.08^‡^
SiO230/2.5	0.05 ± 0.01	0.17 ± 0.07^b^	0.35 ± 0.11	1.12 ± 0.41^B^	0.19 ± 0.08^1^	0.19 ± 0.08^‡^
SiO230/3.5	0.05 ± 0.02	0.18 ± 0.05^b^	0.35 ± 0.17	1.19 ± 0.35^B^	0.23 ± 0.16^1^	0.18 ± 0.11^‡^
SiO2110/2.5	0.05 ± 0.03	0.31 ± 0.10^b^	0.40 ± 0.27	1.93 ± 0.71^B^	0.25 ± 0.22^1^	0.09 ± 0.10^‡^
SiO2110/3.5	0.05 ± 0.01	0.26 ± 0.08^b^	0.37 ± 0.13	1.73 ± 0.40^B^	0.60 ± 0.57^2^	0.54 ± 0.47^∆^

Groups with similar letters or symbols do not present statistical difference.

**Fig 2:**
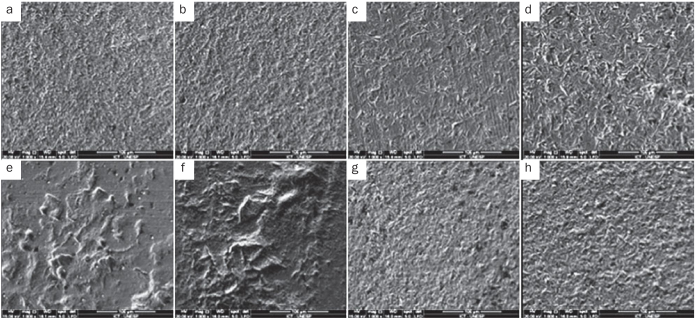
SEM (1000×) of the abraded surfaces of all experimental groups: (a) Al_2_O_3_50/2.5, (b) Al_2_O_3_50/3.5, (c) Al_2_O_3_110/2.5, (d) Al_2_O_3_110/3.5, (e) SiO_2_30/2.5 bar, (f) SiO_2_30/3.5 bar, (g) SiO_2_110/2.5 bar, (h) SiO_2_110/3.5 bar.

**Fig 3:**
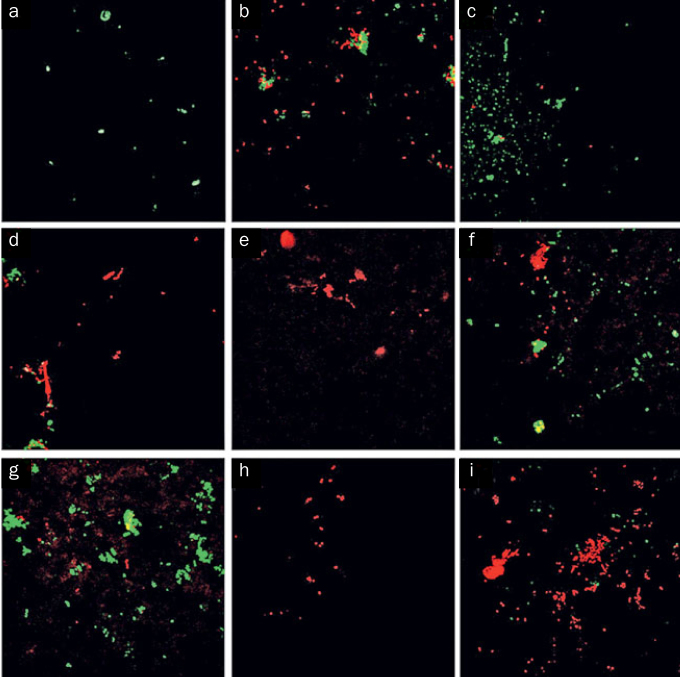
CLSM representative images (63×) for all groups. Viable cells are indicated by green dots. Non-viable cells are indicated by red dots.

The photomicrographs of the surfaces sandblasted with aluminium oxide showed topographical morphology different from that of the surfaces blasted with particles of silica. The surfaces sandblasted with aluminium oxide showed the formation of pits and crevices, which apparently increased in depth as the size of the particle and the pressure increased. Blasting with smaller particles (50 μm) presented a morphological pattern with larger, shallower roughness grooves (Fig 2). When the surfaces abraded with silica were examined, morphology with particle deposition on the surface was observed. When smaller particles (30 μm) were used, deposition occurred, but few cracks formed. The surfaces blasted with larger (110 μm) particles had surface cracks, furrows and increased deposition of particles, resulting in a rougher surface than with the other blasting protocols (Fig 3).

### Biofilm Analysis

The Dunnett test revealed that the group sandblasted with SiO_2_ (110 µm/3.5 bar) showed significantly increased bacterial adhesion (p = 0.002). This group also showed higher biovolume (0.6060 ± 0.57 µm^3^/µm^2^) and average thickness (0.5404 ± 0.48 µm) of bacterial adhesion than the other experimental groups and the control group (Table 2). The representative image is show in Figure 3.

## Discussion

The aim of this study was to evaluate the effects of different sandblasting protocols on surface roughness and initial ‘in situ’ biofilm formation. According to the results, the first hypothesis that the sandblasting favouring early biofilm formation could be denied due to the fact that surfaces with and without sandblasting promoted oral biofilm formation. And the second hypothesis, that larger particles at higher pressure increase the biofilm biovolume and thickness, was partially accepted, because sandblasting with larger particles at higher pressure increased the biofilm biovolume and thickness only for SiO_2_.

There is growing interest in the development of restorative materials with high mechanical strength, clinical longevity, pleasing aesthetics and minimal accumulation of microorganisms on their surfaces. The Y-TZP ceramic meets these requirements for biocompatibility with little accumulation of biofilm.^8,28^ Because of these features, this material can be exposed to the oral environment in the gingival area of the connectors in FPDs.^12^ In addition, some authors have demonstrated that free connectors covered with ceramic and subjected to blasting showed statistically significant improvement in the fracture resistance of FPDs.^30^ However, research has shown that, with blasting as proposed in the literature, surface roughness increases.^24^

Generally, higher surface roughness contributes to bacterial adhesion, because the increasing of surface area.^2,8,21,25^ Also, roughness seems to be more relevant on biofilm formation than surface free energy property.^8^ In addition, the adhesion niches in which bacterial growth occurs protect it from the actions of brushing, muscle activity and salivary flow.^16^ Souza et al^27^ and Özcan et al^20^ reported that when larger particles with higher pressure were applied during the blasting, the surface damage was greater. In the present study, there was no difference between surface roughness; but, in addition, SEM images show that the silica was deposited on the ceramic surface, increasing roughness.^28^ Thus, silica particles with 110 µm and 3.5 bar of pressure promoted a zirconia with rougher surface than when using alumina.^20^ This roughness was associated with a higher surface energy of zirconia surface, and promoted a greater biofilm accumulation. Instead, according to Sato et al,^24^ sandblasting with SiO_2_ and Al_2_O_3_ formed grooves and cavities on zirconia surfaces, although the final roughness did not demonstrate a statistically significant difference, in accordance with the present results. Still there is no consensus in the literature about the effect of sandblasting protocols on the surface topography and early biofilm formation on zirconia.^16,20,28^

The evaluation of roughness was chosen based on the fact that surface texture is important in biofilm formation studies. Previous studies showed that sandblasting on Y-TZP surfaces forms asymmetric peaks and valleys with random impact and promotes a particular surface.^2,6,8,10,11^ In addition, the Ra and Rz parameters are the most used in dental research to express surface differences^6^; however, these parameters reveal only limited information on the characteristics of roughness. The presence of surface defects is camouflaged only when this switch is used. Thus, it is necessary to associate Ra with other parameters for a more realistic picture of surface roughness.^2,8^ Moreover, Rz has the advantage of detecting the presence of peak and valley outliers. When Ra and Rz have similar values, the surface presents a greater uniformity of peaks and valleys. In agreement with the present results, previous studies have shown a discrepancy between Ra and Rz when blasting particles are used.^1,22^ These defects are incorporated into the sandblasted surfaces, originating with sprayed zirconia grains, microcracks or the phase transformation produced by the high energy generated during particle impact, changing the chemical and physical characteristics of Y-TZP surfaces. However, none of these parameters were sufficient to explain the difference in texture that maybe influence in the biofilm formation. Further studies using surface energy analysis and roughness additional parameters should be developed to answer these questions.

CLSM is an alternative tool used in counting biofilms^2,6^ in order to overcome the limitations of scanning electron microscopy. It consists of a non-destructive living cell analysis,^18^ that allows the obtation of three dimensions images of the biofilm. The increase of surface roughness observed in blasted groups did not directly influence initial biofilm formation, represented by the values of biovolume and average thickness. The exception was the group blasted with Rocatec (SiO_2_ = 110 μm) at 3.5 bar pressure, which showed higher biovolume and average thickness when compared with those of the control group. However, the present results are not sufficient to answer the reason that only Rocatec at 3.5 bar significantly increased the biovolume and biofilm thickness once this group presented similar Ra and Rz values than other groups, and other protocols also promoted silica deposition after SiO_2_ abrasion. Most likely, the surface free energy has suffered a modification capable to facilitate biofilm formation. In this way, the authors would like to suggest future papers evaluating the wettability and surface energy of each condition, commonly suggested as related to biofilm formation.^25^

Although studies have shown that exposure of Y-TZP to a humid environment increases the degree of ceramic corrosion, the effects on sandblasted surfaces exposed to the oral environment have not been reported in the literature. New researches correlating the effects of surface treatments on increasing the mechanical strength associated with biofilm accumulation must be performed in the future for a confirmation of the best protocol to be adopted in exposed zirconia of FPDs. As limitations of this study, a short period for biofilm formation was chosen to demonstrate de direct relation of surface condition and the formed biofilm. Also, the purpose of this was not to evaluate different species and quantify them using bacterial colony-forming units, but the authors would like to suggest future investigations on these factors.

## Conclusions

Based on the results of this study, it can be concluded that the air-particle-abrasion protocol with SiO_2_ (110 μm/3.5 bar) should be avoided for the sandblasting of exposed zirconia, because of its increased potential to enhance bacterial adhesion and roughness on zirconia surfaces.
